# Sensitivity to Change of Patient‐Preference Measures for Pain in Patients With Knee Osteoarthritis: Data From Two Trials

**DOI:** 10.1002/acr.22823

**Published:** 2016-07-28

**Authors:** Matthew J. Parkes, Michael J. Callaghan, Terence W. O'Neill, Laura M. Forsythe, Mark Lunt, David T. Felson

**Affiliations:** ^1^ University of Manchester, and the NIHR Manchester Musculoskeletal Biomedical Research Unit, Central Manchester University Hospitals NHS Foundation Trust, Manchester Academic Health Science CentreManchesterUK; ^2^ University of Manchester, and the NIHR Manchester Musculoskeletal Biomedical Research Unit, Central Manchester University Hospitals NHS Foundation Trust, Manchester Academic Health Science Centre, Manchester, UK, and the Salford Royal NHS Foundation TrustSalfordUK; ^3^ University of Manchester, and the NIHR Manchester Musculoskeletal Biomedical Research Unit, Central Manchester University Hospitals NHS Foundation Trust, Manchester Academic Health Science Centre, Manchester, UK, and Boston University School of MedicineBostonMassachusetts

## Abstract

**Objective:**

In osteoarthritis (OA) clinical trials, a pain measure that is most sensitive to change is considered optimal. We compared sensitivity to change of patient‐reported pain outcomes, including a patient‐preference measure (where the patient nominates an activity that aggravates their pain).

**Methods:**

We used data from 2 trials of patients with confirmed (American College of Rheumatology criteria) knee OA: a trial of brace treatment for patellofemoral OA, and a trial of intraarticular steroids in knee OA. Both trials reported an improvement in pain following treatment. Participants rated pain on a 100‐mm visual analog scale (VAS), in the activity that caused them the most knee pain (VAS_NA_), as well as completing questions on overall knee pain and the Knee Injury and Osteoarthritis Outcome Score (KOOS) questionnaire. Western Ontario and McMaster Universities Osteoarthritis Index (WOMAC) scores were also calculated from the KOOS. Standardized changes in each outcome were generated between treatment and control after 6 weeks intervention in the BRACE trial, and 1–2 weeks following intervention in the steroid trial.

**Results:**

The VAS_NA_ produced standardized changes following treatment that were at least as large as other pain outcomes. In the BRACE trial, the between‐groups standardized change with the VAS_NA_ was −0.63, compared with the KOOS pain subscale change of −0.33, and pain in the last week VAS change of −0.56. In the steroid study, within‐group change following treatment in the VAS_NA_ was −0.60, compared to the last week VAS change of −0.51, and KOOS pain subscale change of −0.58.

**Conclusion:**

Pain on nominated activity appears to be at least as, and in some cases more, sensitive to change than the KOOS/WOMAC questionnaire.

## INTRODUCTION

Pain is an outcome that is of particular interest to researchers in osteoarthritis (OA); it is highly clinically relevant and relatively easy to measure. For these reasons, pain is one outcome commonly collected in OA trials. The most commonly used outcome measure is the Western Ontario and McMaster Universities Osteoarthritis Index (WOMAC) 1, 2. However, while data on pain may be collected in most OA clinical trials, the instruments selected to collect such data vary widely 3.

Box 1 **Significance & Innovations**

- To our knowledge, this article is the first to assess the sensitivity of a patient‐preference outcome measure, assessing pain in patients with knee osteoarthritis (OA).
- We find that the sensitivity of this novel outcome is at least comparable, if not marginally superior to, the Knee Injury and Osteoarthritis Outcome Score and the Western Ontario and McMaster Universities Osteoarthritis Index. This is particularly useful, as the novel outcome (the pain on nominated activity visual analog scale, [VAS_NA_]) is only one simple question.
- The VAS_NA_ also allows the collection of data on the distributions of painful activities in different clinical knee OA groups (we compare a mixed knee OA population to a predominantly patellofemoral knee OA population).

Self‐reported pain is often assessed via a single‐item index, e.g., a numerical rating scale (NRS) from 0–10 that rates pain in the last 24 hours. Alternatively, multi‐item questionnaires, such as the WOMAC and Knee Injury and Osteoarthritis Outcome Score (KOOS), can be used; these measures ask several questions on various aspects of pain in order to calculate a composite overall pain score. The questions used in multi‐item measures are typically fixed and inflexible between patients. This rigidity in wording allows the individual patient scores to be compared easily between patients, since they all answer the same question(s). However, there are limitations caused by fixing the question wording in this way. In a multi‐item measure, asking many correlated questions (for example, rating pain in a range of different activities) in an attempt to provide a more comprehensive assessment gathers much more data. However, this is offset by the fact that 1) these questionnaires take longer to administer than a single‐item measure, and 2) there is a risk that many items may be irrelevant to the patient. In theory, irrelevant items will change less following an intervention than more salient ones, and therefore the sensitivity of a tool to detect change will be moderated by the relevancy of the questions asked.

Of single‐item approaches to assessing outcome in OA, an approach that allows for individual variability could be used. Such approaches might ask subjects, for example, to nominate an activity that most aggravates their pain, and then provides a rating score within that context (“Please indicate how much pain you have in that activity”). Current examples of such patient‐preference measures include the McMaster Toronto Arthritis Patient Preference Disability Questionnaire (MACTAR) 4, the Patient‐Specific Index 5, and the Patient‐Generated Index 6. To our knowledge, with the exception of the MACTAR 7, 8, which focuses on patient function and not pain, patient‐preference instruments have not been evaluated in OA. We tested a single‐item version of a patient‐preference questionnaire focused on pain, i.e., the “pain on nominated activity visual analog scale” (VAS_NA_).

Comparisons of outcomes in trials in OA with regards to sensitivity to change 9 have included a mixture of trials, including those reporting a significant change and those that have not. The difficulty in examining trials whereby a treatment effect has not been established is that when an instrument shows poor change following an intervention, it is difficult to distinguish between an instrument with poor responsiveness and a responsive instrument tested when there is no treatment effect. Ideally, the sensitivity to change of outcome measures should be examined in trials showing a treatment effect.

To this end, we sought to characterize and compare pain self‐report outcomes, using data from 2 clinical trials in patients with OA that reported a statistically significant positive change in 1 or more pain outcomes, in an attempt to establish whether there was a difference between single‐ and multi‐item questionnaires, and particularly, whether a patient‐preference approach performed well with respect to sensitivity to change.

## PATIENTS AND METHODS

#### Trials from which data are drawn

Data from 2 interventional clinical trials on patients with knee OA were used in this analysis. In both trials, eligible subjects had to meet American College of Rheumatology criteria for knee OA 10 and have moderate knee pain severity prior to trial commencement. The BRACE trial (ISRCTN 50380458) was a randomized controlled trial targeting persons with painful patellofemoral OA where a patellar knee brace or no brace was administered to participants, and persons were followed for 6 weeks 11.

The TASK (Targeting Synovitis Knee Osteoarthritis) trial (ISRCTN 07329370) was an open‐label trial of persons with painful knee OA monitoring response to intraarticular methylprednisolone. Participants were assessed just prior to treatment and approximately 1 week later 12.

Patients were asked to complete the KOOS questionnaire, as well as 2 single‐item, 100‐mm VAS that rated pain in 2 situations: pain in the last week (VAS_last week_) and pain during an activity nominated by the patient to be the most aggravating for their knee pain (VAS_NA_). The TASK trial participants were additionally asked to score a patient global assessment on a 100‐mm VAS (VAS_global_). All VAS scores ranged from 0–100 (i.e., every millimeter), where 0 indicated no pain at all and 100 indicated pain as bad as the patient could possibly imagine. Both trials have reported a positive effect (i.e., a statistically significant improvement) on pain outcomes.

#### Variable definitions

The KOOS questionnaire is an extension of the WOMAC questionnaire, which features the original WOMAC items plus others. We derived WOMAC scores from the patients’ completed KOOS questionnaires in accordance with the scoring guidelines 13. This therefore meant that both the KOOS and WOMAC were expressed on a 0–100 scale, where 100 indicated no symptoms and 0 indicated extreme symptoms. All other outcomes were completed directly by study participants.

To assess the magnitude of treatment effects, and to allow comparison of effect sizes across outcomes with different scales, we standardized all outcomes, converting them all to have a mean of 0 and an SD of 1. The KOOS features a reversed scoring system, where high scores indicate lower pain rather than higher pain. We reversed the standardized scores of the KOOS outcomes, meaning that greater standardized scores for all outcomes represent increased pain, and lower standardized scores represent decreased pain. Converting the outcomes to standard scores in this way allows all outcomes to be incorporated into 1 common statistical model, which in turn allows for statistical inferences (significance tests) to be made between the outcomes. In contrast, the more commonly used approach of simply deriving effect sizes for each outcome (Cohen's D) only allows comparison of the magnitude of effect sizes without formal statistical tests.

#### Analysis approach

Changes in standardized scores were calculated between the treatment and control groups in the BRACE trial at the 6‐week followup visit. The TASK trial featured an open‐label design without a control group; so the changes in standardized scores for this trial were calculated for the treatment group alone, assessing the change between the baseline and first followup visit after the intervention was applied.

The initial aim was to compare the magnitude of changes in each outcome. We used random‐effects panel linear regression, with the standardized score at the followup visit as the outcome (the 6‐week visit for the BRACE controlled trial, and the postinjection followup visit for the TASK trial), the standardized score at the baseline visit as a covariate, and outcome type (i.e., the scale, a categorical variable, which was one of VAS_NA,_ VAS_last week_, KOOS pain subscale, KOOS symptoms subscale, KOOS activities of daily living subscale, WOMAC pain subscale, WOMAC stiffness subscale, or the WOMAC function subscale, coded as dummy variables) as a predictor variable. The KOOS sport and recreation and quality of life subscales were excluded from analyses, since many participants in both trials left more than 2 items blank (often more), precluding them from being scored in accordance with the KOOS user guide (scoring was available for only 21 of 126 and 106 of 126 in BRACE, and 1 of 127 and 95 of 127 in TASK for sport and recreation and quality of life, respectively). We used participant identifier as the panel variable for the random‐effects model. For the BRACE trial analysis, we also included a predictor variable for treatment group, and an interaction effect between the treatment group and the outcome type.

For the 6‐week treatment versus no treatment comparison, using BRACE data, the full random‐effects model is as follows:

$$y_{ijk}=\mu_{j}\;+\;{\beta_{1}x}_{ij}\;+\;\beta_{jk}\;+\;u_{i}\;+\;W_{ij}$$

Where i = patient, j = outcome (e.g., WOMAC pain) (coded as dummy variables), k = treatment, $y_{ij}$ = standardized score at 6‐week visit (for a given patient and outcome), x = standardized score at baseline, $\beta_{jk}$ = outcome × treatment group interaction,  μ = model intercept, $u_{i}$ = subject‐level random effect, and $W_{ij}$ = error.

The random‐effects model for the TASK data is as follows:

$$y_{ij}=\mu \;+\;X_{ij1}\beta_{1}\;+\;X_{ij2}\beta_{j2}\;+\;u_{i}\;+\;W_{ij}$$

Where $y_{ij}$ = standardized score at the postinjection followup visit, $X_{ij1}$ = standardized score at baseline, $X_{ij2}$ = outcome type (e.g., WOMAC pain) (coded as dummy variables), $u_{i}$ = subject, μ = model intercept, and $W_{ij}$ = error.

The nominated painful activities reported by participants were collected in the form of a free‐text field. We split patients into subgroups based on their nominated activity “themes,” and attempted to match these to WOMAC/KOOS items to better understand the overlap between patient‐preference choices and these instruments.

Statistical analysis was undertaken using Stata (version 13.1). We used a significance level of 5% in all statistical tests.

## RESULTS

#### Demographics

Baseline characteristics of patients from the BRACE and TASK trial were generally similar (Table 1). Patients in TASK were older, with generally more severe OA as seen on radiographs, and had slightly more pain at baseline, which corresponds with the different inclusion criteria of the 2 trials.

**Table 1:** Baseline characteristics of patients from the BRACE and TASK trials^a^

	BRACE(n = 126)		TASK(n = 127)	
Variable	No.	Statistic	No.	Statistic
Age, years	126	55.5 ± 7.5	127	61.98 ± 10.32
Females, frequency (%)	126	72 (57.1)	127	63 (49.6)
BMI, kg/m^2^	126	31.0 ± 5.7	–	–
K/L grade, frequency (% of observations)	88	–	115	–
1		2 (2.3)		0 (0.0)
2		34 (38.6)		43 (37.4)
3		52 (59.1)		64 (55.7)
4		0 (0.0)		8 (7.0)
VAS				
Pain on nominated activity	125	6.5 ± 2.1	122	6.6 ± 1.8
Pain in last week	125	5.9 ± 2.5	124	6.1 ± 2.1
Global pain VAS	–	–	124	4.4 ± 2.3
KOOS subscales				
Pain	126	49.8 ± 18.3	127	45.2 ± 15.1
Symptoms	126	50.2 ± 16.6	126	48.3 ± 16.2
Activities of daily living	126	54.9 ± 20.6	123	49.9 ± 18.1
WOMAC subscales				
Pain	126	55.0 ± 19.9	127	49.8 ± 17.5
Stiffness	126	46.2 ± 20.3	126	39.8 ± 17.2
Function	123	55.6 ± 20.3	119	50.2 ± 18.3

Values are the mean ± SD unless indicated otherwise. Descriptive statistics for the Knee Injury and Osteoarthritis Outcome Score (KOOS), Western Ontario and McMaster Universities Osteoarthritis Index (WOMAC), and visual analog scales (VAS) are presented in their original scales (not standardized) for ease of interpretation. Two patients from the BRACE trial had Kellgren/Lawrence (K/L) grades of 1; they additionally had arthroscopy reports prior to baseline, which confirmed osteoarthritic changes. BMI = body mass index.

#### Comparing change following intervention between outcomes

**Figure 1:**
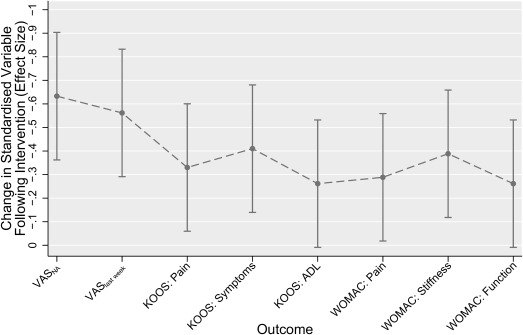
Comparison of standardized change for different outcomes from the BRACE trial. Data depicted refer to the between‐groups difference in the standardized score at the 6‐week followup visit in each outcome, after controlling for baseline score. More negative effect sizes represent larger reductions in pain, and therefore greater sensitivity to change. Error bars indicate 95% confidence intervals for each point estimate. VAS_NA_ = nominated activity visual analog scale; VAS_last week_ = pain last week rated on VAS; KOOS = Knee Injury and Osteoarthritis Outcome Score; ADL = activities of daily living; WOMAC = Western Ontario and McMaster Universities Osteoarthritis Index.

**Figure 2:**
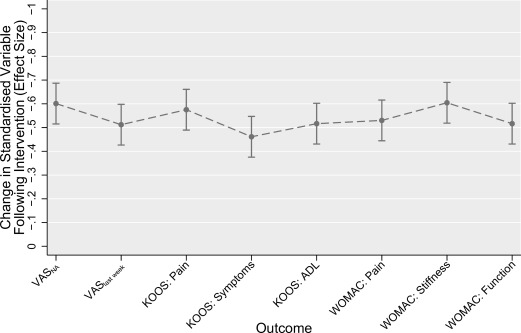
Comparison of standardized response means for different outcomes from the TASK trial. Data depicted refers to the within‐group change in standardized score at the 1‐week followup visit in each outcome, after controlling for baseline score. More negative effect sizes represent larger reductions in pain, and therefore greater sensitivity to change. Error bars indicate 95% confidence intervals for each point estimate. VAS_NA_ = nominated activity visual analog scale; VAS_last week_ = pain last week rated on VAS; KOOS = Knee Injury and Osteoarthritis Outcome Score; ADL = activities of daily living; WOMAC = Western Ontario and McMaster Universities Osteoarthritis Index.

Comparing the postintervention standardized differences across the different outcomes, the VAS_NA_ had the greatest standardized change following treatment in the BRACE study (−0.63) for the between groups analysis (Figure 1). Knee pain in past week (also VAS) also showed a high standardized change (−0.56), and these were higher than the standardized changes for KOOS pain (−0.33) or WOMAC pain (−0.29). Standardized changes appeared more consistent across outcomes in the TASK study than in BRACE. In TASK, the VAS_NA_ tied for the greatest standardized change with the WOMAC stiffness subscale (both had standardized changes of −0.60) (Figure 2). Subsequent pairwise comparisons between outcomes found few significant differences between outcomes (see Supplementary Tables 1 and 2 for the BRACE and TASK trial pairwise comparisons, respectively, available on the *Arthritis Care & Research* web site at http://onlinelibrary.wiley.com/doi/10.1002/acr.22823/abstract).

#### Painful activities nominated by trial participants

A total of 10 specific activity themes were reported by patients in the BRACE and TASK trials (Table 2). Some activities matched activities listed in KOOS and WOMAC, others had no matches, and yet others had unclear matches. “Stairs” was the most often reported activity aggravating participants’ knee pain, and the proportion cited was much greater in BRACE (66.7%), a trial of patellofemoral OA, than in TASK (39.7%). For stair climbing pain, 44% of TASK participants and 88% of the BRACE patients nominated pain either going up or downstairs alone. Patients were asked to nominate an activity without prompting, and we interpreted those patients who cited only one direction when negotiating stairs as having pain that was unidirectional. The KOOS and WOMAC pain questions ask about pain going either up or down stairs, and do not differentiate between directions (Table 3). We performed a further followup analysis that included the KOOS items A1 and A2 (from the function subscale, rather than pain), which do differentiate between direction of stair travel, and found a greater correlation between change in pain on the VAS_NA_ and change in function in these items when the direction of stair travel matched (see Supplementary Tables 3 and 4, available on the *Arthritis Care & Research* web site at http://onlinelibrary.wiley.com/doi/10.1002/acr.22823/abstract). Another frequently nominated painful set of activities was squatting and kneeling, which again is not covered by the KOOS/WOMAC. Five additional themes nominated by participants were clearly not covered by the KOOS or WOMAC, and together these themes made up 30.2% of the BRACE participants’ nominated activities, and 19.0% of the activities of TASK patients (38 and 24 participants, respectively) (Table 2).

**Table 2:** Comparison of painful activities nominated by patients in the BRACE and TASK trials^a^

Nominated (patient‐reported) as most painful activity	BRACE(n = 126)	TASK(n = 126)	Closest‐matched KOOS pain subscale question
Stairs/inclines	84 (66.7)	50 (39.7)	P6, going up or down stairs
Squatting/bending/kneeling	28 (22.2)	7 (5.6)	None^b^
Sitting to standing	7 (5.6)	15 (11.9)	None^b^
Prolonged sitting	3 (2.4)	4 (3.2)	Closest: P8, sitting or lying
Walking	1 (0.8)	33 (26.2)	P5, walking on a flat surface
Walking and stairs	1 (0.8)	1 (0.8)	None
Running	1 (0.8)	1 (0.8)	None
Kneeling and inclines	1 (0.8)	0 (0.0)	None
Turning/twisting	0 (0.0)	5 (4.0)	P2, twisting/pivoting on knee
Standing	0 (0.0)	2 (1.6)	P9, standing upright
Other	0 (0.0)	8 (6.4)	None

a Values are the frequency (% of the total study sample). For simplicity, the nominated activities reported have been collapsed into more general categories (for example, patients reporting “going upstairs” only or “going down inclines or slopes” only are both classified as “stairs/inclines”). “Other” activities reported were “at night” 3, “at work” 1, “dancing” 1, “first thing in morning” 1, “in bed” 1, and “work” 1. One patient did not complete the nominated activity question in the TASK trial at baseline, leaving a total of 126 patients for this outcome. KOOS = Knee Injury and Osteoarthritis Outcome Score.

b Squatting/bending/kneeling could overlap in KOOS with a question of pain when bending and straightening the knee, although our analyses showed little overlap of these elements among patients (those reporting change in pain squatting/bending/kneeling in BRACE did not have similar change in the KOOS question). Pain ongoing from sitting to standing could be mapped to pain with sitting or lying.

**Table 3:** Direction of painful stair travel indicated by patients in the BRACE and TASK trials who nominated “stairs/inclines” as their painful activity^a^

Stair direction declared (if any)	BRACE(total = 84)	TASK(total = 50)
Downstairs only	44 (52.4)	14 (28.0)
Upstairs only	30 (35.7)	8 (16.0)
Bidirectional/not specified	10 (11.9)	28 (56.0)

a Values are the frequency (% of total study sample).

## DISCUSSION

This study attempts to add to the literature on selecting appropriate study outcomes by providing evidence on the sensitivity to change of patient‐reported outcomes in OA trials with a special focus on a patient‐preference measure. Outcomes that are more sensitive to change are good candidates as potential outcomes for future trials. Recent expert consensus meetings have agreed upon the importance of standardizing outcomes in future OA trials, in an attempt to increase validity and sensitivity, and reduce heterogeneity in trial design, and therefore improving the accuracy of future meta‐analyses 14, 15. The present study finds evidence of the VAS_NA_ being more sensitive than other methods, especially in a trial of patients with painful patellofemoral OA.

In both BRACE and TASK, the VAS_NA_, a single question, appeared to have sensitivity to change that at least equaled that of the KOOS and WOMAC subscales, which are a composite of several questions, on a range of activities. This suggests that 1 single question, the “right” question for that patient, is highly sensitive to change. With a fixed single item, the question then becomes “which activity do we choose?”

The approach used by WOMAC/KOOS has a drawback: asking more questions increases the risk of asking unnecessary questions that are irrelevant to a specific patient, and that therefore do not change following intervention. In addition to including possibly irrelevant items, our findings suggest that the KOOS/WOMAC miss items that participants cite as more painful at least in the 2 trials investigated, which suggests that the KOOS/WOMAC may need more items to adequately cover commonly cited painful activities. Alternative approaches with multiple questions exist, such as the Patient‐Reported Outcomes Measurement Information System instruments or instruments using computer adaptive technology (CAT). The premise behind these methodologies 16 is that participants are asked selected, increasingly targeted questions about activity‐related pain from a large bank of questions until a maximal level of precision is attained. While specific WOMAC or KOOS questions may not have relevance to specific patients, we note that both the KOOS and WOMAC were developed after extensive discussions with patients with knee and hip OA to identify activities that were often painful 17, 18.

An alternative approach, used by the VAS_NA_, is to include a free‐text item that allows participants to vary the context of a question. All participants will still score pain throughout the trial, but it allows the participant to give an individualized response on pain in a framework (activity) appropriate and relevant to their situation. Allowing a question to be individualized by the patient may increase sensitivity to change, without adding information on other, less relevant activities.

Asking a single question has other advantages than simply sensitivity/precision. While the activity themes reported in both trials were broadly similar, the proportions in which they were reported differed largely, with BRACE trial participants citing stairs or inclines much more often than those in the TASK trial. This is an unsurprising finding, given that the BRACE trial's inclusion criteria selected persons with patellofemoral OA, whereas the TASK inclusion/exclusion criteria allowed participants with more mixed disease. It also suggests that using fixed instruments like the WOMAC and KOOS for patellofemoral OA is likely to compromise sensitivity to change, whereas this choice may be more reasonable in trials of knee OA in general.

There are a number of possible advantages to this more bottom‐up approach of involving patients’ perspectives directly in outcome measures in OA, as opposed to the top‐down method of the researcher deciding which questions are best for the patient. It includes more of a patient's view directly in the study. Furthermore, it provides additional data alongside a simple pain score, as patients also provide qualitative data on the sorts of activities that are painful to them, i.e., activities that might not otherwise have been considered. For example, we found many patients in both the BRACE and TASK trials appear to find either going up or going downstairs alone more aggravating to their pain, which contrasts with many pain questionnaires that ignore the direction of stair travel. A potential drawback to this approach is that while it aims to maximize sensitivity to detect a treatment effect, by selecting questions for activities that are most painful, it risks ignoring those activities that are most important to the patient's activities of daily living.

Our analysis also included as a by‐product an examination of the comparative sensitivity to change of a global knee pain question (the VAS_last week_) versus the WOMAC/KOOS pain scale. For the patellofemoral OA trial, the VAS_last week_ was more sensitive to change than the KOOS/WOMAC, but that was not the case for the TASK study. The difference in VAS_last week_ responsiveness between TASK and BRACE is an interesting finding, and the reasons for this observation are unclear. One possible explanation for this difference could be the different samples used in each of the trials. The TASK trial was comprised of subjects with mixed OA (not one compartment specifically), unlike BRACE, which selected patients with patellofemoral OA only. The KOOS and WOMAC were specifically designed for use in mixed disease contexts, and it may be that the patellofemoral patients are not captured quite as well, hence the difference between the 2 trials. Given that the other outcomes are fairly consistent between BRACE and TASK, another possible explanation is linked to the focus of BRACE on patellofemoral OA versus TASK, which recruited a more general group of patients with painful knee OA. Our data do not permit us to conclude which of these alternative choices is likely to be consistently more sensitive to change in OA trials. Others have reported that a global pain question in a fixed timeframe is more sensitive to change than the WOMAC pain subscale 19, 20 and other “complex” multi‐item measures 21. The same trend has also been noted when comparing a global function question versus the WOMAC function subscale, with the single‐item global question having superior sensitivity to change 22. In contrast, Dworkin et al in a meta‐analysis combining many single fixed item indices of pain found that the WOMAC produced a greater standardized change 23. This analysis, however, combined many indices of pain and, as noted by the authors, it is unclear whether the heterogeneity of both the included trials, and the combination of outcomes used, may have contributed to this conflicting trend.

This study is not without limitations. We specifically selected only 2 studies for this analysis. It would be advantageous to conduct this analysis across a greater number of trials to confirm whether the trends we observed are consistent across a range of trial types and OA populations. However, an analysis of sensitivity to change is best conducted on a trial whereby the researcher is (at least) reasonably sure that a true treatment effect has occurred. It is difficult to selectively search for OA trials that are 1) positive, i.e., observed a true pain reduction, 2) in a mixture of OA subpopulations, and 3) collected data and reported on multiple collinear outcomes. Our study is at least informative in part, since our analysis used one trial focused on patellofemoral OA and the other unselected knee OA patients.

The analysis approach we used allowed us to test for differences between outcomes. While we did observe some statistically significant differences between outcomes, no one outcome was clearly superior in both trials in terms of sensitivity to change (see Supplementary Tables 5 and 6, available on the *Arthritis Care & Research* web site at http://onlinelibrary.wiley.com/doi/10.1002/acr.22823/abstract). This is not an unexpected finding, given that all measures should theoretically measure the same construct (pain, in this study), and should therefore have at least similar effect sizes. Given the similarity of these measures, it would require either large differences in effects, or large sample sizes to establish differences in outcomes. The trials we analyzed were neither designed nor powered to observe such small differences between outcome measures, and therefore the likelihood of observing truly significant differences was unfortunately lacking. In the future, we would recommend performing this type of analysis in larger real‐world trials. Another potential limitation is that use of a single question focused on pain with 1 activity may compromise content validity, the evaluation of all of the impacts of a disease.

If a patient nominates an activity in which pain is ameliorated completely following treatment, then that activity experiences floor effects, especially in a long‐term study. For example, a treatment that fully cures pain from sitting to standing after initial application would register no change at the subsequent followup, highlighting the importance of the activity that the patient selects. In a similar vein, in longer‐term followup, activities that were selected by participants at baseline may become less relevant as the pattern of disability changes. Allowing the patient to alter the selected activity should it reach the minimal score is complex, particularly if patients select an activity that is not in the same “dimension.” For example, a patient nominates “pain when getting up from sitting,” then improves in the trial, and then switches to “pain when doing exercise classes.” These 2 activities are acceptable on their own, at each visit, but it is unclear how comparable they are on a unidimensional scale, which is a limitation of the proposed flexible approach. CAT methods sidestep this issue through prior calibration of item‐bank questions to ensure that they all measure 1 common metric. A drawback of the VAS_NA_ used in BRACE and TASK is that it is susceptible to floor effects when used in a trial that 1) has a large pain effect, and 2) is very long term; these are not uncommon properties of OA trials.

The VAS_NA_ uses a visual analog score to collect continuous data on pain. Some groups have reported that participants find VAS difficult to understand, leading to decreased response rates when compared to a Likert scale or NRS 24. Indeed, some trials have investigated this issue as a primary study aim 25. Perhaps in the future, following the recommendations of the Initiative on Methods, Measurement, and Pain Assessment in Clinical Trials group 9, the VAS_NA_ could be applied using an NRS (in effect, an NRS_NA_), which would have the advantages of both sensitivity and increased response rates.

In conclusion, we suggest that in knee OA studies patient‐preference instruments may offer sensitivity to change and the opportunity to detect treatment effects that might be missed by conventional fixed instruments. Our work needs to be corroborated in other studies.

## AUTHOR CONTRIBUTIONS

All authors were involved in drafting the article or revising it critically for important intellectual content, and all authors approved the final version to be submitted for publication. Mr. Parkes had full access to all of the data in the study and takes responsibility for the integrity of the data and the accuracy of the data analysis.

**Study conception and design**. Parkes, Felson.

**Acquisition of data**. Callaghan, O'Neill, Forsythe.

**Analysis and interpretation of data**. Parkes, Lunt, Felson.

## Supporting information

Supplementary Materials
