# Supplementary material for: Sensitivity to Change of Patient‐Preference Measures for Pain in Patients With Knee Osteoarthritis: Data From Two Trials
Source: Arthritis Care Res (Hoboken). 2016 Jul 28;68(9):1224–31. doi: 10.1002/acr.22823 (PMC5025729; doi:10.1002/acr.22823)
Supplement: Supplementary file 1 — Supplementary Materials [file ACR-68-1224-s001.docx]

**Detailed Results from the Standardised Change Analysis**

**Supplementary Table 1: Pairwise comparison of BRACE study standardised changes.**

| **Outcome** | **VAS_NA_** | **VAS_late week_** | **KOOS: Pain** | **KOOS: Symptoms** | **KOOS: ADL** | **WOMAC: Pain** | **WOMAC: Stiffness** | **WOMAC: Function** |
| --- | --- | --- | --- | --- | --- | --- | --- | --- |
| **VAS_NA_** |  |  |  |  |  |  |  |  |
| **VAS_last week_** | 0.071  (-0.192 to 0.334),  *0.596* |  |  |  |  |  |  |  |
| **KOOS: Pain** | 0.303  (0.040 to 0.566),  *0.024* | 0.232  (-0.032 to 0.495),  *0.084* |  |  |  |  |  |  |
| **KOOS: Symptoms** | 0.223  (-0.040 to 0.486),  *0.097* | 0.152  (-0.111 to 0.415),  *0.258* | -0.080  (-0.343 to 0.183),  *0.551* |  |  |  |  |  |
| **KOOS: ADL** | 0.371  (0.108 to 0.635),  *0.006* | 0.300  (0.037 to 0.563),  *0.025* | 0.069  (-0.194 to 0.332),  *0.609* | 0.149  (-0.115 to 0.412),  *0.268* |  |  |  |  |
| **WOMAC: Pain** | 0.344  (0.081 to 0.608),  *0.010* | 0.273  (0.010 to 0.536),  *0.042* | 0.042  (-0.221 to 0.305),  *0.757* | 0.122  (-0.142 to 0.385),  *0.365* | -0.027  (-0.290 to 0.236), *0.840* |  |  |  |
| **WOMAC: Stiffness** | 0.245  (-0.019 to 0.508),  *0.068* | 0.173  (-0.090 to 0.436),  *0.196* | -0.058  (-0.321 to 0.205),  *0.664* | 0.022  (-0.241 to 0.285),  *0.871* | -0.127  (-0.390 to 0.136), *0.345* | -0.100  (-0.363 to 0.163), *0.457* |  |  |
| **WOMAC: Function** | 0.371  (0.108 to 0.635), *0.006* | 0.300  (0.037 to 0.563), *0.025* | 0.069  (-0.194 to 0.332), *0.609* | 0.149  (-0.115 to 0.412), *0.268* | 0.000  (-0.263 to 0.263), *1.000* | 0.027  (-0.236 to 0.290), *0.840* | 0.127  (-0.136 to 0.390), *0.345* |  |

**Key to table format:**

| Difference in standardised change  (95 confidence interval for change)  *p-value* |
| --- |

**Supplementary Table 2: Pairwise comparison of TASK study standardised changes**

| **Outcome** | **VAS_NA_** | **VAS_late week_** | **KOOS: Pain** | **KOOS: Symptoms** | **KOOS: ADL** | **WOMAC: Pain** | **WOMAC: Stiffness** | **WOMAC: Function** |
| --- | --- | --- | --- | --- | --- | --- | --- | --- |
| **VAS_NA_** |  |  |  |  |  |  |  |  |
| **VAS_last week_** | 0.089  (-0.032 to 0.210), *0.151* |  |  |  |  |  |  |  |
| **KOOS: Pain** | 0.026  (-0.095 to 0.147), *0.676* | -0.063  (-0.184 to 0.058), *0.308* |  |  |  |  |  |  |
| **KOOS: Symptoms** | 0.140  (0.018 to 0.261), *0.024* | 0.051  (-0.070 to 0.172), *0.411* | 0.114  (-0.007 to 0.235), *0.066* |  |  |  |  |  |
| **KOOS: ADL** | 0.085  (-0.037 to 0.206), *0.172* | -0.004  (-0.126 to 0.117), *0.944* | 0.059  (-0.063 to 0.180), *0.343* | -0.055  (-0.177 to 0.066), *0.372* |  |  |  |  |
| **WOMAC: Pain** | 0.071  (-0.050 to 0.192), *0.252* | -0.018  (-0.139 to 0.103), *0.772* | 0.045  (-0.076 to 0.166), *0.466* | -0.069  (-0.190 to 0.052), *0.266* | -0.014  (-0.135 to 0.108), *0.826* |  |  |  |
| **WOMAC: Stiffness** | -0.003  (-0.125 to 0.118), *0.956* | -0.092  (-0.214 to 0.029), *0.136* | -0.029  (-0.151 to 0.092), *0.636* | -0.143  (-0.265 to -0.022), *0.021* | -0.088  (-0.209 to 0.033), *0.155* | -0.074  (-0.196 to 0.047), *0.229* |  |  |
| **WOMAC: Function** | 0.085  (-0.037 to 0.206), *0.172* | -0.004  (-0.126 to 0.117), *0.944* | 0.059  (-0.063 to 0.180), *0.343* | -0.055  (-0.177 to 0.066), *0.372* | 0.000  (-0.121 to 0.121), *1.000* | 0.014  (-0.108 to 0.135), *0.826* | 0.088  (-0.033 to 0.209), *0.155* |  |

**Key to table format:**

| Difference in standardised change  (95 confidence interval for change)  *p-value* |
| --- |

**Supplementary Table 3: Stair use analysis. Spearman’s correlations between baseline to 6-week follow-up change in VAS_NA_ score, KOOS overall score, and individual KOOS items, in subgroups of patients nominating pain aggravated when going downstairs only, upstairs only, or either direction/unclear – BRACE study.**

| **BRACE Study** | | **Nominated Activity Category** | | | |
| --- | --- | --- | --- | --- | --- |
|  |  | Downstairs only | Upstairs only | [Both directions/ Unclear] | [All Stairs] |
| No. of patients reporting this nominated activity, with complete KOOS data | | 37 | 22 | 7 | 66 |
| **Trial Outcome** | | **Correlation between change in the VAS_NA_,**  **and change in the following outcomes (r_s_)** | | | |
| Pain on Nominated Activity (VAS_NA_) | | 1.00 | 1.00 | 1.00 | 1.00 |
| Overall KOOS Pain Subscale Score | | 0.32 | 0.34 | -0.49 | 0.26 |
| KOOS P1 | Pain frequency. | 0.13 | -0.04 | -0.41 | 0.02 |
| KOOS P2 | Twisting/pivoting on knee. | 0.27 | 0.42 | -0.44 | 0.23 |
| KOOS P3 | Extending knee fully. | 0.15 | 0.27 | 0.80 | 0.16 |
| KOOS P4 | Bending knee fully. | 0.25 | 0.36 | -0.56 | 0.25 |
| KOOS P5 | Walking on a flat surface. | 0.11 | 0.11 | -0.65 | 0.14 |
| KOOS P6 | Going up or down stairs. | **0.33** | **0.21** | **-0.96** | **0.21** |
| KOOS P7 | At night while in bed. | -0.06 | 0.01 | -0.31 | -0.07 |
| KOOS P8 | Sitting or lying. | -0.01 | 0.32 | -0.17 | 0.17 |
| KOOS P9 | Standing upright. | 0.16 | 0.31 | -0.46 | 0.18 |
| KOOS A1 | Function, descending stairs. | **0.51** | **0.13** | -0.72 | **0.35** |
| KOOS A2 | Function, ascending stairs. | **0.23** | **0.71** | -0.85 | **0.37** |

*The above table is a complete case analysis: the N for the ‘all stairs’ group is lower than in Table 2 in the manuscript,*

*since some patients had missing responses in either the baseline or follow-up visit for the individual KOOS items included above.*

**Supplementary Table 4: Stair use analysis. Spearman’s correlations between baseline to follow-up change in VAS_NA_ score, KOOS overall score, and individual KOOS items, in subgroups of patients nominating pain aggravated when going downstairs only, upstairs only, or either direction/unclear – TASK study.**

| **TASK Study** | | **Nominated Activity Category** | | | |
| --- | --- | --- | --- | --- | --- |
|  |  | Downstairs only | Upstairs only | [Both directions/ Unclear] | [All Stairs] |
| No. of patients reporting this nominated activity, with complete KOOS data | | 19 | 8 | 35 | 62 |
| **Trial Outcome** | | **Correlation between change in the VAS_NA_,**  **and change in the following outcomes (r_s_)** | | | |
| Pain on Nominated Activity (VAS_NA_) | | 1.00 | 1.00 | 1.00 | 1.00 |
| Overall KOOS Pain Subscale Score | | 0.65 | 0.78 | 0.77 | 0.78 |
| KOOS P1 | Pain frequency. | 0.49 | 0.20 | 0.67 | 0.54 |
| KOOS P2 | Twisting/pivoting on knee. | 0.55 | 0.40 | 0.41 | 0.42 |
| KOOS P3 | Extending knee fully. | 0.40 | 0.73 | 0.64 | 0.63 |
| KOOS P4 | Bending knee fully. | 0.29 | 0.43 | 0.61 | 0.52 |
| KOOS P5 | Walking on a flat surface. | 0.40 | 0.72 | 0.56 | 0.55 |
| KOOS P6 | Going up or down stairs. | **0.55** | **0.85** | **0.65** | **0.66** |
| KOOS P7 | At night while in bed. | 0.58 | 0.76 | 0.71 | 0.71 |
| KOOS P8 | Sitting or lying. | 0.40 | 0.64 | 0.62 | 0.60 |
| KOOS P9 | Standing upright. | 0.79 | 0.68 | 0.52 | 0.60 |
| KOOS A1 | Function, descending stairs. | **0.81** | **0.88** | 0.72 | **0.76** |
| KOOS A2 | Function, ascending stairs. | **0.64** | **0.94** | 0.68 | **0.71** |

*The above table is a complete case analysis: the N for the ‘all stairs’ group is lower than in Table 2 in the manuscript,*

*since some patients had missing responses in either the baseline or follow-up visit for the individual KOOS items included above.*

**Supplementary Table 5: BRACE study standardised change following intervention (see figure 1 in article).**

| **Outcome** | **Standardised change following treatment (95% CI)** | **p** | **Groups** | | |  |  |
| --- | --- | --- | --- | --- | --- | --- | --- |
| VAS_NA_ | -0.633 (-0.903 to -0.362) | <0.001 | A |  |  |  |  |
| VAS_last week_ | -0.562 (-0.832 to -0.291) | <0.001 | A | B |  |  |  |
| KOOS: Pain | -0.330 (-0.600 to -0.060) | 0.017 |  | B | C |  |  |
| KOOS: Symptoms | -0.410 (-0.681 to -0.139) | 0.003 | A | B | C |  |  |
| KOOS: ADL | -0.261 (-0.532 to 0.009) | 0.058 |  |  | C |  |  |
| WOMAC: Pain | -0.288 (-0.559 to -0.018) | 0.037 |  |  | C |  |  |
| WOMAC: Stiffness | -0.388 (-0.659 to -0.118) | 0.005 | A | B | C |  |  |
| WOMAC: Function | -0.261 (-0.532 to 0.009) | 0.058 |  |  | C |  |  |
| *‘Groups’ column classifies outcomes into groups whereby the observed standardised change does not differ at the 95% level. For example, the standardised change for VAS_NA_ did not differ significantly from the change observed for the VAS_last week_,KOOS symptoms subscale, and WOMAC stiffness subscale, at the 95% level. Outcomes with more than one letter had change that was not isolated to one ‘group’ at the 95% level. For more detail on specific pairwise comparisons, refer to eTable 3. Data shown refers to the between-groups difference in standardised score at the 6-week follow-up visit, in each outcome, after controlling for baseline score.* | | | | | | |  |

**Supplementary Table 6: TASK study standardised change following intervention (see figure 2 in article).**

| **Outcome** | **Standardised change following treatment (95% CI)** | **p** | **Groups** | | |  |  |
| --- | --- | --- | --- | --- | --- | --- | --- |
| VAS_NA_ | -0.601 (-0.687 to -0.515) | <0.001 | A |  |  |  |  |
| VAS_last week_ | -0.512 (-0.598 to -0.426) | <0.001 | A | B |  |  |  |
| KOOS: Pain | -0.575 (-0.661 to -0.489) | <0.001 | A | B |  |  |  |
| KOOS: Symptoms | -0.461 (-0.547 to -0.375) | <0.001 |  | B |  |  |  |
| KOOS: ADL | -0.516 (-0.602 to -0.431) | <0.001 | A | B |  |  |  |
| WOMAC: Pain | -0.530 (-0.616 to -0.444) | <0.001 | A | B |  |  |  |
| WOMAC: Stiffness | -0.604 (-0.690 to -0.519) | <0.001 | A |  |  |  |  |
| WOMAC: Function | -0.516 (-0.602 to -0.431) | <0.001 | A | B |  |  |  |
| *‘Groups’ column classifies outcomes into groups whereby the observed standardised change does not differ at the 95% level. For example, the standardised change for VAS_NA_ did not differ significantly from the change observed for all outcomes with the exception of the KOOS symptoms subscale, at the 95% level. Outcomes with more than one letter had change that was not isolated to one ‘group’ at the 95% level. For more detail on specific pairwise comparisons, refer to eTable 4. Data shown refers to the between-groups difference in standardised score at the 6-week follow-up visit, in each outcome, after controlling for baseline score.* | | | | | | |  |
